# Hepatic Portal Venous Gas (HPVG) after Ingestion of Chlorine Bleach: A Transient Phenomenon

**DOI:** 10.3390/diagnostics13243615

**Published:** 2023-12-07

**Authors:** Francesco M. Arico, Francesco Buemi, Pietro Pitrone, Claudio Giardina, Renato Trimarchi, Flavia Borruto, Sarah Doria, Cristina Turiaco, Simona Caloggero

**Affiliations:** 1 Diagnostic and Interventional Radiology Unit, BIOMORF Department, University Hospital “Policlinico G. Martino”, 98124 Messina, ME, Italy; 2 Radiology Unit, “Papardo” Hospital, 98158 Messina, ME, Italy; 3 Department of Radiology, ASST Bergamo Ovest, Ospedale Treviglio-Caravaggio, 24047 Treviglio, BG, Italy

**Keywords:** HPVG, portal pneumatosis, caustic ingestion, bleach ingestion, sodium hypochlorite, computed tomography, spiral, diagnostic techniques

## Abstract

We present a case involving a 32-year-old man who ingested chlorine bleach with self-defeating intent. The ingestion of bleach can lead to a wide range of consequences, from mild mucosal burns to severe complications, rarely resulting in death. This case highlights the association between chlorine bleach ingestion and the development of hepatic portal venous gas (HPVG), a radiological finding traditionally thought to carry poor prognoses. The HPVG in this case resolved spontaneously within 24 h with conservative management, indicating its transient nature. The exact pathophysiological mechanisms responsible for HPVG after the ingestion of toxic substances .remain only partially understood. One hypothesis suggests that extensive damage to the gastrointestinal wall caused by caustic agent may allow enteric gas to enter the portal system. While HPVG after toxic ingestion is often transient, its consequences and potential risks should be carefully considered. Hyperbaric oxygen therapy is suggested in cases with neurological symptoms. In conclusion, HPVG is not a specific disease but rather a manifestation of various underlying factors, and its development in the context of chlorine bleach ingestion represents an additional insight to its understanding. It can be associated with severe medical conditions, but it is also found in less severe cases that can be managed conservatively.

**Figure 1:**
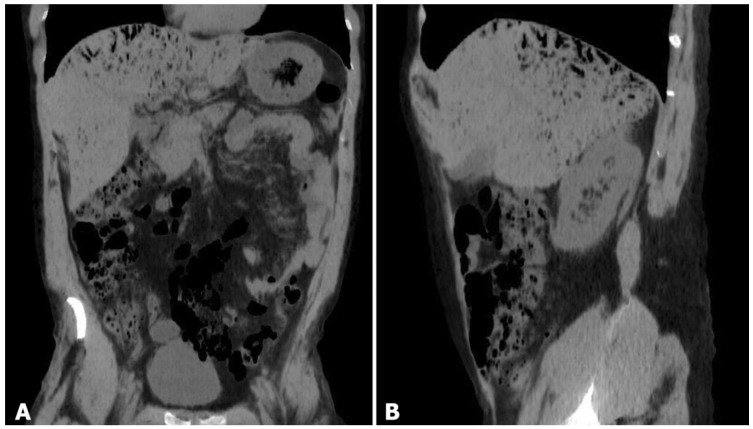
A 32-year-old man with a history of psychiatric illness was brought to Emergency Department (ED) after the ingestion of two sips (approximately 30 mL) of household bleach containing sodium hypochlorite with self-defeating intent. On arrival, he presented with abdominal pain and vomiting. There were no specific clinical findings and no neurological abnormality. The patient underwent routine blood test examinations, electrocardiogram (EKG), non-contrast thoraco-abdominal CT scan and upper endoscopy. Laboratory tests and the EKG were substantially normal. The abdominal unenhanced CT scan (**A**,**B**) revealed the presence of a large amount of air content within the liver, with peripheral and subcapsular distribution predominantly in the upper and posterior segments. Additionally, a diffuse edema of the gastric wall and duodenum was observed. Significantly, there was no evidence of free intraperitoneal air. Traditionally, hepatic portal venous gas (HPVG) (Figure 1) was considered a radiological sign of poor prognosis. It is characterized by the presence of air content in the portal tree within the liver, with a typical peripheral distribution (Figure 2) [1]. Nowadays, severe pathologies are diagnosed at earlier stages thanks to technological advancements, allowing for timely treatment and a reduction in mortality rates. On the other hand, the improvement in diagnostic capabilities have made the presence of HPVG more common in radiology. Therefore, HPVG alone no longer represents a surgical indication, and the choice of treatment primarily depends on the underlying medical condition and the patient’s clinical status. HPVG is a radiological sign that can be found across a spectrum of various pathologies, ranging from more severe cases requiring immediate surgical intervention to instances where it appears as a transient phenomenon likely to be resolved with conservative therapy [2]. Various medical conditions have been linked to the presence of HPVG in imaging results, such as bowel ischemia, necrotizing enterocolitis, inflammatory bowel diseases, massive gastric dilatation, and septic conditions, including acute diverticulitis, acute appendicitis, acute cholangitis, acute necrotizing pancreatitis and emphysematous infectious spondylodiscitis [3,4,5]. In addition to these rather common causes, other causes related to invasive iatrogenic procedures have been associated with HPVG; these include extracorporeal shock wave lithotripsy, gastro-jejunal anastomotic leakage after laparoscopic gastric bypass, percutaneous endoscopic gastrostomy tube placement, endoscopic biliary sphincterotomy, gastroscopic biopsy and umbilical vein catheterization [6,7,8,9]. Idiopathic causes were identified in 15% of cases [3]. Finally, there is growing scientific evidence for the presence of transient HPVG after the ingestion of toxic agents, including hydrogen peroxide-based agents, oil painting solvents or pharmaceutical overdose [10,11,12,13,14].

**Figure 2:**
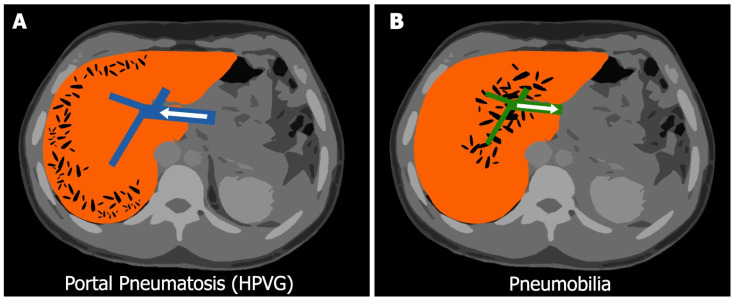
The graphic representation shows the main differential diagnosis of HPVG (**A**) with pneumobilia (**B**), with which it can often be mistaken. They are two distinct radiological signs, each one with its unique characteristics that can be differentiated through CT imaging. HPVG, also called portal pneumatosis, manifests as small, comma-shaped tubular areas of decreased attenuation within the liver, with sub-capsular and peripheral distribution. This phenomenon occurs due to the accumulation of gas within the intrahepatic portal veins where it is carried by centrifugal blood flow towards the hepatic periphery. Figure 2A shows in blue a schematic representation of the portal system with the flow directed to the periphery [3]. Pneumobilia refers to the presence of air within the biliary tree. In cases of pneumobilia, the air is centrally distributed, and it does not extend to within 2 cm of the liver capsule. This central distribution of the air, expression of hepatofugal flow of bile within the biliary system towards the hepatic hilum, is a key characteristic that helps differentiate it from HPVG. In Figure 2B shows in green a schematic representation of the bile ducts with hepatofugal flow. Furthermore, in pneumobilia, a confluence of air is often observed also in the common hepatic duct. Both HPVG and pneumobilia show more frequent left-lobe predilection [14]. We describe the case of a 32-year-old man with a history of psychiatric illness who presented to the Emergency Department (ED) after the ingestion of two sips (approximately 30 mL) of household hypochlorite sodium-based bleach, also called chlorine bleach, with self-defeating intent. The consequences of chlorine bleach ingestion depend mainly on the concentration of NaOCL present in the solution and on the total amount ingested. Nausea, vomiting, a burning sensation in the mouth, first-degree burns with hyperemia and edema of the gastroesophageal mucosa are the most common consequences after the ingestion of a solution of less than 6% sodium hypochlorite concentration. However, serious complications such as stenosis, strictures, or gastroesophageal perforations occur when sodium hypochlorite is ingested in large amounts (5 mL/kg in children, and 150–200 mL in adults) or at concentrations more than 6% [15]. Disastrous consequences resulting in death have been rarely described [16]. Bleaching agent ingestion represents a notable public health concern compared to other chemical agents, not only for the consequences of the ingestion itself but also for the widespread availability as an over-the-counter product. Bleaching agents intended for disinfectant purposes typically contain a <6% solution of NaOCL. While the precise concentration of the bleach used in our case remains unreported, considering the minimal organic damage observed, we speculate it may be a formulation with a concentration below 6%. Our case describes for the first time the association between chlorine bleach ingestion and the development of HPVG. This radiological finding disappeared spontaneously within 24 h with conservative management, demonstrating its transient nature (Figure 3A,B). There have been numerous pieces of evidences of HPVG emerging after the ingestion of toxic substances, among which oxygen peroxide stands out in terms of frequency. In most of the cases, rapid resolution after conservative therapy has been documented [10,11,12,13,14]. The concentration as well as the amount of the substance ingested are important prognostic factors; this must be determined by asking the patient or a family member as this information can help predict the severity of the event. In a simplified approach, it may be advisable to solicit either the patient or their relatives for a photograph of the substance label in use. This would facilitate the extraction of comprehensive information, encompassing details such as brand, sodium hypochlorite concentration, pH, and other pertinent factors. However, the exact pathophysiological mechanisms responsible for this phenomenon remain only partially elucidated. One possible hypothesis is that extensive damage to the gastrointestinal wall, resulting from the contact with the caustic agent, could promote the entry of enteric gas into the capillary veins by an impaired epithelial barrier, and thus accumulate in the portal system [17]. Despite the transient manifestation of HPVG observed in analogous cases and the documented instances managed through non-operative approaches, a comprehensive understanding of the repercussions of this phenomenon remains elusive. Furthermore, there exists a potential risk of neurological or cardiac sequelae in selected patients, emphasizing the need for vigilance in this regard [18]. Hyperbaric oxygen (HBO) therapy is suggested as a prudent measure by many authors to prevent the further progression of gas emboli, and it should be considered in the presence of neurological symptoms. The choice to initiate this treatment should be based on a thoughtful evaluation of the accessibility of suitable services and a careful assessment of the treatment’s potential advantages and disadvantages [18,19].

**Figure 3:**
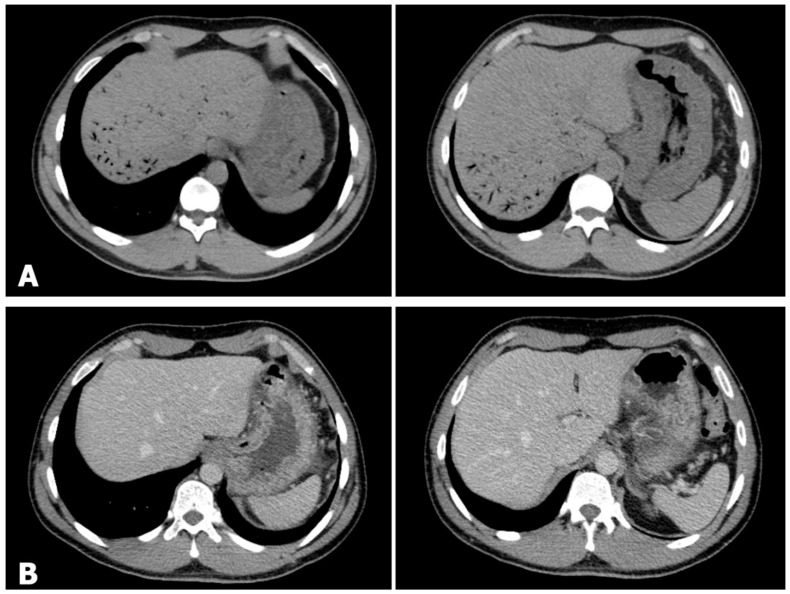
Considering the patient’s favorable clinical status and the absence of any radiological signs of perforation, a conservative approach was chosen. The patient was maintained on a fasting regimen and intravenous fluids and proton pump inhibitors were administered. On the day following admission, a comprehensive clinical assessment, encompassing a thorough examination of the abdomen and neurological function, yielded normal results. An upper endoscopy was also performed, confirming caustic agent damage of the gastric mucosa (Figure 4). Subsequently, a follow-up abdominal CT scan performed after the administration of intravenous contrast medium was carried out to re-evaluate the initial imaging observations (**A**). The follow-up scan (**B**) showed the spontaneous resolution of hepatic portal venous gas (HPVG) and the ongoing presence of the previously noted gastric and duodenal wall abnormalities.

**Figure 4:**
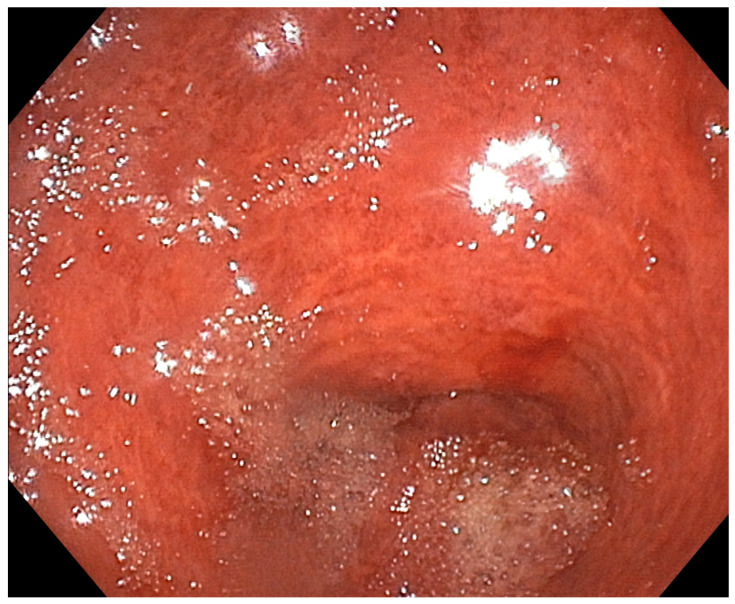
Esophagogastroduodenoscopy performed 24 h after the admission revealed gastric lesions resulting from caustic ingestion. Endoscopic visualization of the gastric antrum revealed mild mucosal damage, superficial erosions and hemorrhage (Zargar IIA). By the end of the third day, the patient was transferred to the psychiatric department. In conclusion, HPVG is a radiological sign that may manifest in the context of various underlying conditions. Although the exact pathophysiological mechanism is not entirely clear, in our case, it is plausible to assume that the caustic damage caused by the contact of the solution containing NaOCl with the gastro-intestinal mucosa resulted in the passage of gas into the portal system. While this finding has traditionally demonstrated its association with clinical conditions necessitating immediate intervention, numerous scenarios exist in which it serves as a “benign” sign, correlated with a mortality rate approaching 0% [17]. Its development subsequent to the ingestion of chlorine bleach, as well as other toxic substances, constitutes instances where its management could be conservative. Therefore, HVPG should not autonomously dictate clinical or surgical interventions; emphasis should be placed on evaluating the severity of the underlying disease for informed decision making in the realm of management strategies.

## Data Availability

Data are contained within the article.
